# A study of the impact of project-based learning on student learning effects: a meta-analysis study

**DOI:** 10.3389/fpsyg.2023.1202728

**Published:** 2023-07-17

**Authors:** Lu Zhang, Yan Ma

**Affiliations:** ^1^Institute of Computer and Information Science, Chongqing Normal University, Chongqing, China; ^2^Institute of Smart Education, Chongqing Normal University, Chongqing, China

**Keywords:** project-based learning, learning effects, 21st century skills, higher-order thinking, meta-analysis

## Abstract

**Introduction:**

With the educational reform for skills in the 21st century, a large number of scholars have explored project-based learning. However, whether project-based learning can effectively improve the learning effect of students has not yet reached a unified conclusion.

**Method:**

This study uses a meta-analysis method to transform 66 experimental or quasi-experimental research papers based on project-based learning over the past 20 years into 190 effect values from the sample size, mean, and standard deviation of experimental data during their experiments, and to conduct in-depth quantitative analysis.

**Results:**

The results of the study showed that compared with the traditional teaching model, project-based learning significantly improved students’ learning outcomes and positively contributed to academic achievement, affective attitudes, and thinking skills, especially academic achievement.

**Discussion:**

The results of the moderating effects test indicated that the effectiveness of project-based learning and teaching was influenced by different moderating variables, including country region, subject area, type of course, academic period, group size, class size, and experimental period : (1) from the perspective of country geography, the effects of project-based learning in Asia, especially in Southeast Asia, were significantly better than those in Western Europe and North America; (2) in terms of curriculum, project-based learning promotes student learning effects more significantly in engineering and technology subjects, and is better applied in laboratory classes than in theory classes; (3) from a pedagogical point of view, project-based learning is more suitable for small group teaching, in which the group size is 4-5 people teaching the best results; (4) in view of the experimental period, 9-18 weeks is more appropriate and has more obvious advantages for application at the high school level.

## 1. Introduction

Project-based learning (PBL) is a new model of inquiry-based learning that is centered on the concepts and principles of a subject, with the help of multiple resources and continuous inquiry-based learning activities in the real world, with the aim of producing a complete project work and solving multiple interrelated problems within a certain period of time (Jingfu and Zhixian, 2002). s a new student-centered teaching approach, project-based learning directly points to the goal of cultivating 21st-century skills, especially higher-order thinking skills, and higher-order thinking occurs based on problem-solving, a challenging problem that emphasizes real-world situations and open environments, and project-based learning motivates students to continuously explore in the process of problem-solving, thus promoting the development of higher-order thinking.

In the era of digital transformation of education, the new generation of information technologies such as artificial intelligence, big data, and metaverse are bringing great changes to education at an unimaginable speed, and at the same time posing unprecedented challenges to talent training. Cultivating students with higher-order thinking skills that can adapt to the future development of society and reasonably cope with the complex real world has become an important mission in the current education reform and development around the world (Ma and Yang, 2021). Different types of problems produce different teaching methods and also guide the development of students’ different thinking skills. Project-based learning, as a new type of teaching and learning method in the context of curriculum and teaching reform, takes real life as the background, is driven by practical problems, breaks the disciplinary boundaries, integrates multiple disciplines into one project, and develops students’ future-oriented abilities——creative thinking, problem raising, problem solving, critical thinking, communication and collaboration, etc. The advantages of this approach over traditional teaching and learning models are being recognized and explored. A large number of studies on the effects of project-based learning have been done, but there is not complete agreement on the effects on the development of students’ thinking skills, academic performance, and affective attitudes.

Over the past few decades, project-based learning has received a lot of attention in the field of education. Many studies have shown that project-based learning can improve students’ learning motivation, problem-solving skills, teamwork, and communication skills. However, due to the complexity and diversity of project-based learning, as well as differences in research methods, research findings on its effectiveness and influencing factors vary. A key research question in project-based learning meta-analytic studies is to assess the impact of project-based learning on student learning outcomes, including student performance in the areas of academic achievement, thinking skills, and affective attitudes. By combining the results of multiple independent studies, more accurate and reliable conclusions can be obtained to further understand the effects of project-based learning. In addition, project-based learning meta-analysis studies can help reveal the factors and mechanisms influencing project-based learning. By comparing the learning effects under different project-based learning conditions, researchers can analyze the impact of factors such as project characteristics, instructional design, and learning environment on student learning. This can help guide the design and implementation of project-based learning and promote effective student learning. Based on this, this study compensates for the limitations of individual studies by integrating and synthesizing multiple independent studies in order to systematically assess the effects of project-based learning, provide more accurate and reliable evidence, and reduce the chance of research findings. At the same time, project-based learning meta-analysis can provide a broader perspective to help researchers and educational policy makers gain a comprehensive understanding of the effects and influencing factors of project-based learning, so that they can develop more effective teaching strategies and policies to promote the improvement and development of project-based learning.

## 2. Literature review and theoretical framework

One view is that project-based learning can significantly improve student learning outcomes, including academic achievement, motivation, and higher-order thinking skills. Karpudewan et al. (2016) explored the feasibility of improving energy literacy among secondary school students using a project-based instructional approach. The quantitative results of the study showed that students exposed to a PBL curriculum had better performance on energy-related knowledge, attitudes, behaviors, and beliefs. The quantitative results of the study showed that students exposed to the PBL curriculum outperformed students taught using the traditional curriculum. The quantitative results of the study showed that students exposed to the PBL course outperformed students taught with traditional courses in terms of energy-related knowledge, attitudes, behaviors, and beliefs. The results of Zhang Ying’s intrinsic motivation scale, which was administered to 21 private university students before and after they received project-based learning, showed that there were significant differences in students’ interest, autonomy, and competence before and after, which positively influenced students’ intrinsic motivation to learn (Zhang, 2022). Yun (2022) used the fifth-grade project “Searching for Roots. Xu Hui Yuan” project-based learning as an example to discuss that project-based in-depth ritual education can develop students’ core literacy. Biazus and Mahtari (2022) conducted a quasi-experiment using project-based learning and direct instructional learning models and found that the PBL model had a significant impact on the enhancement of creative thinking skills of secondary school students. Parrado-Martínez and Sánchez-Andújar (2020) explored the effects of project-based learning on ninth-grade students’ writing skills and found that cooperative work in project-based learning potentially promoted students’ critical thinking, communication, and collaboration skills, significantly improving middle school students’ English writing skills. Hernández-Ramos and De La Paz (2009) found that students in project-based learning conditions showed significant improvements in content knowledge measures and growth in their historical thinking skills compared to students in control schools. Most researchers agree that STEM as a form of project-based learning and STEM integration will have a positive impact on education, with the advantages outweighing the disadvantages (Hamad et al., 2022; Wardat et al., 2022).

Another view is that project-based learning has the same effect or even some negative effects compared to traditional instruction. García-Rodríguez et al. (2021) conducted an intervention experiment in undergraduate education to test the effectiveness of a student-centered project-based learning approach in promoting student skill acquisition. The study found that students’ problem-solving and information management skills, two instrumental general competencies were not improved. The results of ÇAKICI’s project-based learning activities on fifth-grade children’s science achievement showed that although project-based activities significantly improved children’s science achievement, attitudes toward science did not change. Gratchev and Jeng (2018) explored whether the combination of traditional teaching methods and project-based learning activities improved students’ learning experiences, and data collected over 3 years showed that the two groups’ achievements were very similar, and the findings indicated that students were less motivated to accept new learning methods such as PBL. Parrado-Martínez and Sánchez-Andújar (2020) found that the implementation of PBL did not significantly change students’ perceived utility of teamwork, communication, and creativity. Kızkapan and Bektaş (2017) examined the effects of project-based learning and traditional learning methods on the academic performance of seventh graders, and the results showed no significant differences between the experimental and control groups on post-test “achievement test” scores. Sivia et al. (2019) used a mixed triangulation-convergence approach to examine the difference in student engagement between project-based and non-project-based learning units and found that project-based learning did not significantly increase student engagement. Karaçalli and Korur (2014) used a quasi-experimental design to teach the experimental group using a project-based learning approach, and the results showed no statistically significant effect on students’ attitudes toward learning across groups.

In summary, a review of the literature reveals that the research findings and teaching effectiveness of project-based learning have not yet been uniformly determined, and few studies have systematically analyzed and evaluated the optimal group size, class size, curriculum type, and subject area of project-based learning. Therefore, based on 66 empirical research papers that conducted experimental or quasi-experimental studies on project-based learning and traditional teaching, this study quantifies the true magnitude of the impact of the project-based learning approach on students’ learning outcomes and seeks to summarize the experience of applying project-based learning in schools in order to provide a reference for developing project-based teaching. And an attempt is made to answer the following research questions:

1. Does project-based learning significantly improve students’ thinking skills, academic performance, and affective attitudes compared to traditional teaching methods?
2. How do different moderating variables (type of course, learning section, group size, class size, subject category, experiment period, country region.) affect students’ learning effects?

**Figure 1:**
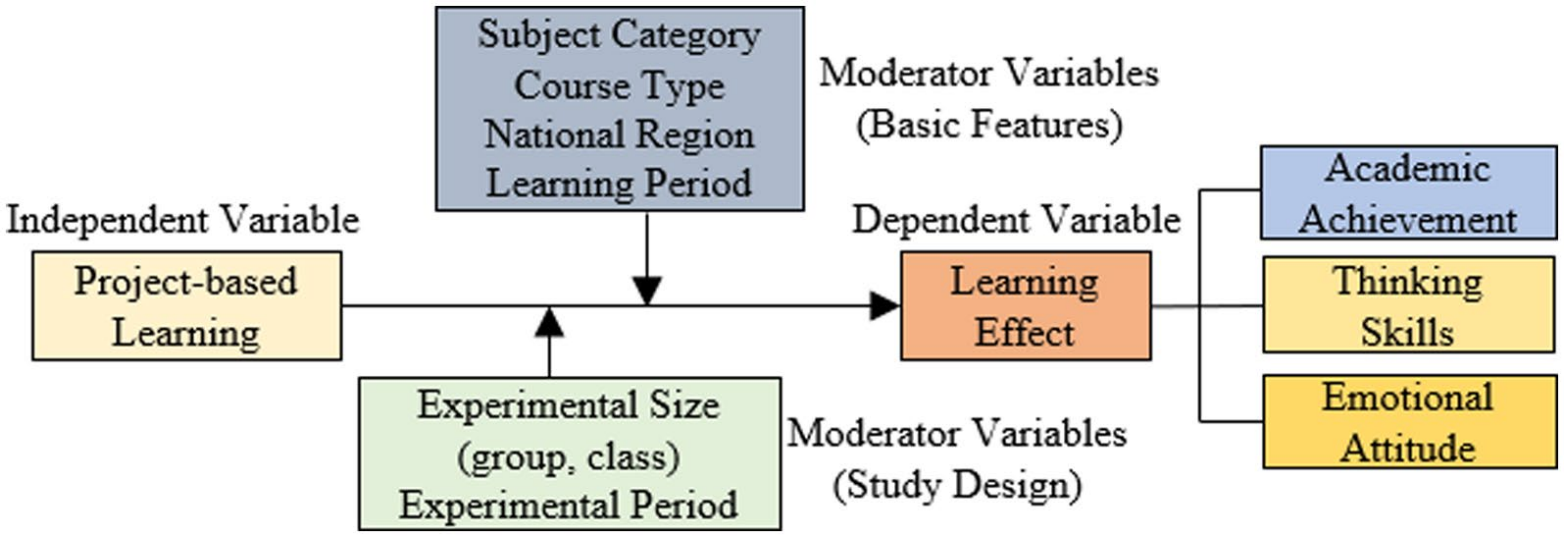
Research framework diagram.

Since the purpose of this study was to explore the effect of project-based learning on learning effectiveness and to explore other factors that may moderate this effect. Therefore, based on relevant research findings on the effect of project-based integrated learning on learning effectiveness and the results of literature coding, the meta-analytic theoretical framework for this study, as shown in Figure 1.

## 3. Study design

### 3.1. Methods

Meta-Analysis is a quantitative analysis method that extracts and organizes multiple results of experimental or quasi-experimental studies on the same research question and then produces an average effect value by weighting the sample size, standard mean deviation, and other data from the existing research results and analyzes the effect value to obtain the results. The meta-analysis method has been widely used in education. This study compares and combines literature on the same research topic but with different research results by extracting data such as pre and post-test means, sample sizes, and standardized mean differences from relevant literature, while using the standard deviation (SMD), which can correct for small sample bias, as the effective value to indicate the degree of influence of project-based instruction on student learning outcomes. The study entered the relevant data into CMA meta-analysis software (Comprehensive Meta Analysis 3.0) for data analysis.

### 3.2. Research process

To ensure the quality of the study, this study strictly followed the meta-analysis criteria proposed by Glass (1976), which was mainly divided into four assessment procedures: literature collection, literature coding, effect size calculation, and moderating variable analysis, and finally a comprehensive effect size exploration and study results.

#### 3.2.1. Literature search

To ensure the timeliness of the study, this study mainly searched the relevant research on the topic of project-based learning since 2003 to 2023, mainly in CNKI, Springer Link, Web of Science, Semantic Scholar and other databases, and searched the literature by “AND” or “OR” logical word collocation of project-based learning and learning effectiveness keywords. The keywords of project-based learning include: project-based learning, PBL, project teaching; the keywords of learning effect include: learning effect, learning performance, learning achievement, learning*, learning outcome, learning result, etc. And the selected articles are all from SSCI or SCI authoritative journals, Chinese core journals of article literature type and part of the master’s degree thesis. To avoid omissions, this study also supplemented the search with the references of relevant articles.

#### 3.2.2. Literature selection and inclusion criteria

**Figure 2:**
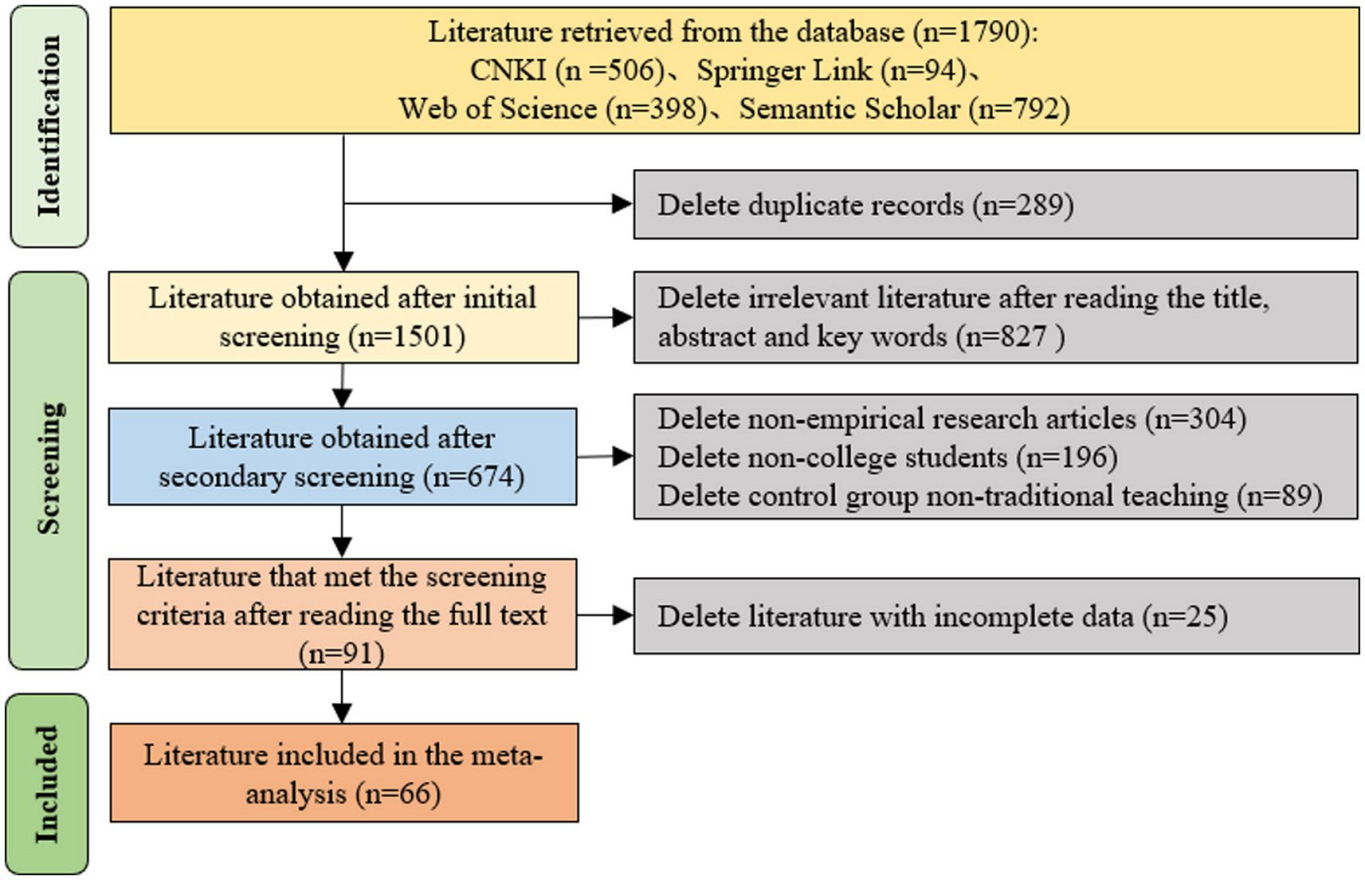
Flow chart of literature screening.

To find articles that meet the subject matter requirements, this study used the (Page, 2021) process for literature processing (Vrabel, 2009), the literature search, screening, and inclusion process is shown in Figure 2. Combining the needs of the meta-analysis method itself and ensuring the accuracy and rigor of the research results, the following selection and inclusion criteria were used: (1) duplicate literature had to be removed; (2) it had to be a study of the effects of project-based learning versus traditional teaching models on learning effectiveness; (3) it had to be an empirical research type article; (4) complete data that could calculate the effect values had to be available. A total of 91 articles were screened by two researchers in the inclusion phase, and those with inconsistent screening were discussed, and the final decision was made to include 66 articles in the meta-analysis, which met the inclusion criteria for the number of articles in the meta-analysis method.

#### 3.2.3. Literature code

The concept of project-based learning was first introduced by American educator William Heard Kilpatrick proposed (Kilpatrick, 1918). In the 1920s and 1930s, project-based learning was widely used in the lower grades of elementary and secondary schools in the United States; in 1969, McMaster University in Canada officially launched the PBL teaching model within the school. To compare the variability of the effects of project-based learning in countries around the world, the regions of the countries where the study was conducted were coded and divided into North America, Oceania, Southeast Asia, and other regions. As project-based learning is used more frequently in the classroom, whether there is an ideal group size to facilitate student learning outcomes (Wei et al., 2020), and the impact of group size on academic achievement (Al Mulhim and Eldokhny, 2020), which academic section, subject, and course type is better taught, are questions that should be addressed. Therefore, the coding of this study included the following seven main items: subject category, course type, country region, academic section, class size, group size, and experimental period, and categorized learning outcomes into three main categories: academic achievement, thinking skills, and emotional attitudes. Because this study included 66 documents with 190 effect sizes, only part of the feature coding content is displayed, as shown in Table 1 (Kelly and Mayer, 2004; Mioduser and Betzer, 2007; Hernández-Ramos and De La Paz, 2009; Domínguez and Elizondo, 2010; Keleşoğlu, 2011; Çakici and Türkmen, 2013; Karaçalli and Korur, 2014; Bilgin et al., 2015; Astawa et al., 2017; Kızkapan and Bektaş, 2017; ShiXuan, 2017; Yuan, 2017; Praba et al., 2018; Yexin, 2019; Faqing, 2020; Gao, 2020; Lei, 2020; Ling, 2020; Linxiao, 2020; Lu, 2020; Luo, 2020; Mingquan, 2020; Rui, 2020; Yanan, 2020; Yang, 2020; Akharraz, 2021; Cong, 2021; Migdad et al., 2021; Xiaolei, 2021; Wang, 2021a,b, 2022; Jina, 2022; Ma, 2022; Xu, 2022; Xuezhi, 2022; Yating, 2022; Ying, 2022; Yuting, 2022; Zhang, 2022). To ensure the objectivity of the coding process, this study was completed independently by two researchers for the 66 empirical research articles included in the meta-analysis, and the coding results were tested for consistency using SPSS 24.0, and the Kappa value was 0.864, which was greater than 0.7, indicating that the coding effect was valid and the results were credible.

**Table 1:** Code list (due to space limitation, only part of the coding content is shown).

Number	Author (year)	Learning effect		Study design				Basic features		
				Group size	Class size	Experimental period	Learning Section	Subject category	Course types	Country region
1	Duman and Yavuz (2018)	Emotional attitude	Learning attitude	4–5	Small	1–8 weeks	High school	Humanities and social	theory	Western Asia
2	Zulaeha and Marpaung (2020)	Academic achievement	Academic achievement	4–5	Small	1–8 weeks	University	Humanities and social	theory	Southeast Asia
3	1 Parrado-Martínez and Sánchez-Andújar (2020)	Emotional attitude	Self-efficacy	8 and above	Middle	1–8 weeks	Junior high school	Humanities and social	experimental	Oceania
4	2 Parrado-Martínez and Sánchez-Andújar (2020)	Emotional attitude	Learning attitude	8 and above	Middle	1–8 weeks	Junior high school	Humanities and social	experimental	Oceania
5	3 Parrado-Martínez and Sánchez-Andújar (2020)	Thinking skills	Cooperation ability	8 and above	Middle	1–8 weeks	Junior high school	Humanities and social	experimental	Oceania
6	4 Parrado-Martínez and Sánchez-Andújar (2020)	Thinking skills	Problem raising	8 and above	Middle	1–8 weeks	Junior high school	Humanities and social	experimental	Oceania
7	5 Parrado-Martínez and Sánchez-Andújar (2020)	Academic achievement	Academic achievement	8 and above	Middle	1–8 weeks	Junior high school	Humanities and social	experimental	Oceania
8	6 Parrado-Martínez and Sánchez-Andújar (2020)	Academic achievement	Academic achievement	8 and above	Middle	1–8 weeks	Junior high school	Humanities and social	experimental	Oceania
9	1 Kaldi et al. (2011)	Emotional attitude	Self-efficacy	8 and above	Small	1–8 weeks	Primary school	Natural science	theory	Western Europe
10	2 Kaldi et al. (2011)	Thinking skills	Cooperation ability	8 and above	Small	1–8 weeks	Primary school	Natural science	theory	Western Europe
11	3 Kaldi et al. (2011)	Academic achievement	Academic achievement	8 and above	Small	1–8 weeks	Primary school	Natural science	theory	Western Europe
12	WEN-JYE. (2009)	Academic achievement	Academic achievement	8 and abovec	Small	Over 18 weeks	University	Engineering and technology	experimental	East Asia
13	1 Safa. (2021)	Emotional attitude	Self-efficacy	4–5	Small	1–8 weeks	University	Humanities and social	theory	Western Asia
14	2 Safa. (2021)	Thinking skills	Decision-making ability	4–5	Small	1–8 weeks	University	Humanities and social	theory	Western Asia
15	Kızkapan and Bektaş (2017)	Academic achievement	Academic achievement	8 and above	Small	1–8 weeks	Junior high school	other	theory	Western Asia

#### 3.2.4. Data analysis

Based on the completion of the literature coding, the calculation of the effect size (Standardized difference in means), including sample size, standard deviation, and mean value, was performed by finding the relevant experimental data in the literature. The effect size values were calculated as follows:

Starting with Mean, SD, N in each group.

Raw difference in means.

RawDiff = Mean1-Mean2.

SDP = Sqr (((N1–1) * SD1^2 + (N2-1) * SD2^2)/(N1 + N2–2))).

Standardized difference in means.

StdDiff = RawDiff/SDP.

The next stage was data analysis by (1) publication bias test. A funnel plot was used for qualitative analysis, while a combination of Begg’s rank test and loss of safety coefficient was used for quantitative analysis; (2) Heterogeneity test. The aim was to determine whether there was heterogeneity among the samples in this study; (3) Calculation of effect size values. To quantify the degree of influence of project chemistry learning on learning outcomes; (4) the moderating variables were tested. All data analyses in this study were conducted using Comprehensive Meta Analysis 3.0.

## 4. Results

### 4.1. General effect size results

#### 4.1.1. Publication bias test

**Figure 3:**
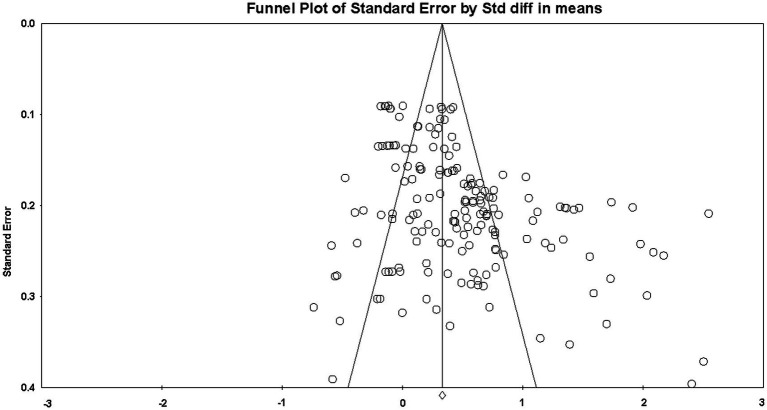
Publication bias funnel plot.

In this study, the std. diff in means (SMD) value was selected as the unbiased effect value, and also to ensure the possibility that the results reported in the literature do not deviate from the true results, the publication bias was analyzed qualitatively using funnel plots, and the publication bias was analyzed qualitatively using Begg’s rank test, Trim and Fill and Fail-safe N to quantitatively analyze publication bias. Publication bias is critical to the results of meta-analysis, and if the research literature is not systematically representative of all existing research in the field in general, it indicates that publication bias may exist (Higgins and Thompson, 2002). As shown in Figure 3, the majority of study effect values were clustered within the funnel plot, and a small number of effect values were relative to the right, with Begg’s rank test *Z* = 5.082 > 1.960 (*p* < 0.05), indicating a possible publication bias. Therefore, the severity of publication bias was further identified using the loss of safety factor, which showed *N* = 2,546, much larger than “5K + 10” (*K* = 190), suggesting that an additional 2,546 unpublished studies would be required to reverse the results (Rothstein et al., 2006), and it can be concluded that there is no significant publication bias in this study.

#### 4.1.2. Heterogeneity test

To ensure that the effect values of the independent samples in this study are combinable, Q and I2 values were used to define heterogeneity. Higgins et al. classified heterogeneity as low, medium, or high, as measured by the magnitude of the I2 statistic, which was 25, 50, and 75%, respectively. In addition, if the Q statistic is significant then the hypothesis that there is no heterogeneity among the sample data should be rejected. Based on the forest plot of I2 = 87.4% > 50% and *Q* = 1496.2 (*p* < 0.001), the results indicate that there is a high degree of heterogeneity between the samples, therefore, this study used a random effects model for correlation analysis to eliminate some of the effects of heterogeneity, and also further indicates that it is necessary to conduct a moderated effects test to examine the effect of project-based learning on learning effects.

### 4.2. Results about problem of studies’ fields

#### 4.2.1. The overall impact of project-based learning on student learning outcomes

Cohen (1988) proposed the effect value analysis theory in 1988, he believed that the effect standard measure effect is determined by the effect value (ES), when the ES is less than 0.2, it means that there is a small effect impact, when the ES is between 0.2–0.8 means that there is a moderate effect, when the ES > 0.8 means that there is a significant effect impact. This study included 190 experimental data from 66 empirical research papers, and as shown in Table 2, the combined effect value of the impact of project-based learning on student learning outcomes was 0.441, close to 0.5 and *p* < 0.001, indicating that project-based learning has a large degree of impact on learning outcomes and is an effective teaching approach.

**Table 2:** Main effects test.

Effect model	Number of effects	Effect value	95% confidence interval		Two-tailed test	
			Lower limit	Upper limit	Z-value	*p*-value
Fixed effects	190	0.306	0.453	0.331	24.854	0.000
Random effects	190	0.441	0.371	0.551	12.334	0.000

In this study, the literature included in the meta-analysis was divided into three subcategories of academic achievement, thinking skills, and emotional attitudes according to the “three-dimensional goals” for analysis. Moderately positive impact (SMD = 0.650), and the total effect values for affective attitudes and thinking skills were 0.389 and 0.386, respectively.

Based on the deeper connotation of “three-dimensional goals,” this study classifies affective attitudes into learning motivation, learning attitude, learning interest, and self-efficacy; thinking skills into creative thinking ability, computational thinking ability, decision-making ability, critical thinking ability, problem-solving ability, problem raising ability, collaboration ability, and comprehensive application ability. As shown in Table 3. In terms of affective attitudes, project-based learning influenced more on students’ interest in learning (SMD = 0.713), and also had moderate positive effects on learning motivation (SMD = 0.401) and learning attitudes (SMD = 0.536), with lower effects on self-efficacy; in terms of thinking skills, project-based learning had the most significant effects on students’ creative thinking skills (SMD = 0.626) and computational thinking skills (SMD = 0.719) had the most significant effect, followed by problem solving, collaboration, and general application skills, but the effects on decision making, critical thinking, and problem raising skills did not reach a statistically significant level.

**Table 3:** Effects of project-based learning on different learning outcomes.

Learning effect	Number of effects	Total effect value	Type	Number of effects	effect value	95% confidence interval		Two-tailed test	
						Lower limit	Upper limit	*Z*-value	*P*-value
Academic achievement	40	0.650	Academic achievement	40	0.650	0.453	0.846	6.480	0.000
Emotional attitude	50	0.389	Learning motivation	5	0.401	0.162	0.640	3.283	0.001
			Learning attitude	18	0.536	0.312	0.759	4.702	0.000
			Learning interest	7	0.713	0.282	1.145	3.242	0.001
			Self-efficacy	20	0.181	0.062	0.301	2.969	0.003
Thinking skills	100	0.386	Creative thinking skills	14	0.626	0.288	0.965	8.956	0.000
			Computational thinking skills	5	0.719	0.332	1.106	3.645	0.000
			Decision-making skills	9	0.092	−0.127	0.311	0.820	0.412
			Critical thinking skills	2	0.512	−0.583	1.607	0.916	0.360
			Problem-solving skills	18	0.365	0.182	0.548	3.909	0.000
			Problem-raising ability	13	0.117	−0.092	0.326	1.096	0.273
			Collaboration ability	17	0.404	0.141	0.666	3.016	0.003
			Comprehensive application ability	22	0.432	0.217	0.646	3.945	0.000

#### 4.2.2. Examining the effects of different moderating variables on student learning

First, in terms of country region as a moderating variable, the overall effect value of its moderating effect on learning effectiveness was 0.358 and *p* < 0.001, indicating a moderate effect and the effects varied across countries. In terms of effect values between groups, although project-based learning originated in the United States and was first applied in American countries such as Canada, its effect on student learning outcomes was not significant (SMD = 0.061, *p* = 0.429 > 0.05), and there was no significant difference in whether or not project-based learning was used; instead, the application of project-based learning produced better learning outcomes in Asian countries, especially in Southeast Asian countries (SMD = 0.684), followed by West Asia (SMD = 0.594).

Second, looking at the school level as the moderating variable, the overall effect value SMD = 0.355, in order of effect value from smallest to largest, is university (SMD = 0.116) < junior high school (SMD = 0.520) < primary school (SMD = 0.527) < high school (SMD = 0.720), which indicates that there are differences in the effects of project-based learning on the learning outcomes of students in different school levels, with the effects on high school, primary school, and junior high school, while the effect on college was relatively small.

Third, using group size as the moderating variable, the combined effect value of group size on learning effectiveness is 0.592 (*p* < 0.001), which is close to 0.6, indicating that the effect of group size on students’ learning effectiveness is more significant and has a moderate to a high degree of facilitating effect. In terms of the effect values of different sizes, the effect values are all positive, indicating that the group learning style is effective and has different degrees of facilitating effects on learning effects, with the most significant facilitating effect of a group size of 4–5 students on learning effects (SMD = 0.909).

Fourth, to test the applicability of project-based learning on different class sizes, the class sizes were divided into three sizes according to the sample size: small (1 ~ 100 students), medium (100 ~ 200 students), and large (200 ~ 300 students), and the data in Table 4 show that the overall effect value of the moderating effect of class size on the learning effect is 0.378, *p* < 0.001, indicating that project-based learning on different class size. Looking specifically at each size, the degree of impact was higher for small class sizes (SMD = 0.483), followed by medium size (SMD = 0.466), but lower and not significant for large class sizes (SMD = 0.106, *p* = 0.101 < 0.05).

**Table 4:** Results of moderating effects of different moderating variables.

Adjustment variables	Type	Number of effects	Effect value	Degree of influence	95% confidence interval		Two-tailed test	
					Lower limit	Upper limit	*Z*-value	*P*-value
Country Region	North Africa	1	2.033	Larger	0.190	0.857	1.249	0.212
	North America	14	0.061	Smaller	1.447	2.618	6.806	0.000
	Oceania	9	0.334	Middle	0.058	0.258	1.239	0.215
	Southeast Asia	15	0.684	Middle	0.019	0.184	1.586	0.113
	Eastern Europe	1	0.305	Middle	0.382	0.665	7.229	0.000
	East Asia	94	0.550	Middle	0.509	0.710	11.850	0.000
	South America	1	1.145	Larger	0.190	0.857	1.249	0.212
	Western Europe	32	0.076	Smaller	1.447	2.618	6.806	0.000
	Western Asia	23	0.594	Middle	0.058	0.258	1.239	0.215
	Combined effect volume	190	0.358	Middle	0.303	0.420	12.073	0.000
Learning period	Primary School	41	0.527	Middle	0.369	0.684	6.541	0.000
	Junior High School	52	0.520	Middle	0.388	0.651	7.754	0.000
	High School	40	0.720	Middle	0.558	0.881	8.727	0.000
	University	57	0.116	Smaller	0.027	0.204	2.550	0.011
	Combined effect volume	190	0.355	Middle	0.294	0.417	11.293	0.000
Group size*	3 and below	2	0.778	Middle	0.464	1.093	4.852	0.000
	4–5	23	0.909	Larger	0.720	1.099	9.428	0.000
	6–7	42	0.436	Middle	0.310	0.562	6.764	0.000
	8 and above	14	0.514	Middle	0.229	0.799	3.538	0.000
	Combined effect volume	81	0.592	Middle	0.498	0.686	12.344	0.000
Class size	small	104	0.483	Middle	0.387	0.578	9.936	0.000
	Middle	71	0.466	Middle	0.338	0.594	7.124	0.000
	Big	15	0.106	Smaller	0.021	0.233	1.642	0.101
	Combined effect volume	190	0.378	Middle	0.313	0.444	11.319	0.000
Subject category	Engineering and technology	62	0.619	Middle	0.482	0.756	8.844	0.000
	Humanities and Society	55	0.284	Middle	0.166	0.402	4.720	0.000
	Life science	3	0.708	Middle	0.482	0.933	6.147	0.000
	Natural science	42	0.484	Middle	0.365	0.602	7.997	0.000
	Other	28	0.275	Middle	0.107	0.442	3.214	0.001
	Combined effect volume	190	0.443	Middle	0.380	0.506	13.773	0.000
Course type	Theory class	103	0.393	Middle	0.297	0.488	8.088	0.000
	Experimental class	87	0.498	Middle	0.395	0.600	9.541	0.000
	Combined effect volume	190	0.441	Middle	0.372	0.511	12.420	0.000
Experimental period	Single experiment	28	0.359	Middle	0.241	0.478	5.947	0.000
	1–8 weeks	62	0.498	Middle	0.380	0.615	8.321	0.000
	9–18 weeks	35	0.673	Middle	0.494	0.853	7.353	0.000
	18 weeks or more	65	0.300	Middle	0.179	0.420	4.874	0.000
	Combined effect volume	190	0.424	Middle	0.360	0.488	12.972	0.000

^*^To avoid the inclusion of literature that did not provide group size numbers from influencing the study results, only literature that did provide data was included in the analysis here.

Other literature included in the meta-analysis (Beier et al., 2019; Castro-Vargas et al., 2020; Chung, 2021; Ergül and Kargın, 2014; Hugerat, 2016; Hung et al., 2012; Lazić et al., 2021; Lin and Lu, 2018; Mark, 2022; Ozdamli and Turan, 2017; Ruiz-Rosa et al. (2013; Wang et al., 2016; Wurdinger and Qureshi, 2015; Zulaeha et al., 2020.).

Fifth, when subject categories were viewed as moderating variables, all subject effect values were larger than 0, with a combined effect value of SMD = 0.443 (*p* < 0.001), suggesting that project-based learning had a positive degree of enhancement on learning effectiveness across subjects, reaching a statistically significant difference. Due to the relatively small amount of literature in other categories and life sciences, this study focuses on the effects of project-based learning on learning outcomes in engineering and technology, humanities and social, and natural sciences. In each of the subjects, Engineering and Technology (SMD = 0.619) > Natural Sciences (SMD = 0.484) > Humanities and Society (SMD = 0.284), the results indicate that project-based learning has the most significant impact on learning effectiveness in Engineering and Technology and relatively less in Humanities and Society.

Sixth, the overall effect value SMD = 0.441 when looking at the type of course as a moderating variable, while the between-group effect test between experimental and theoretical classes reached a statistically significant level (*p* < 0.001). The effect of project-based learning on student learning outcomes was more pronounced in experimental classes (SMD = 0.498), which was greater than the overall combined effect value, consistent with the finding that project-based learning is more suitable and effective teaching strategy for engineering and technology disciplines, while the use of project-based teaching in theory classes (SMD = 0.393) was below the average effect value.

Seventh, in terms of the experimental period as a moderating variable, there were significant differences in project-based learning across experimental periods (*p* < 0.001), with a moderating overall effect value of SMD = 0.424. The best effect of instructional facilitation was observed for the duration of 9–18 weeks (SMD = 0.673), which was better than single experiments (SMD = 0.359) and 1–8 weeks (SMD = 0.498), with a relatively weak effect on learning outcomes beyond 18 weeks (SMD = 0.3000).

## 5. Discussion

This study used meta-analysis to systematically review and quantitatively analyze 66 experimental or quasi-experimental research papers published between 2003 and 2023 on the effects of project-based instruction on student learning, and to dissect the differences brought about by different moderating variables. The results show that: ① project-based learning can significantly improve students’ learning outcomes compared with traditional teaching models; ② the effects of project-based teaching and learning are influenced by different moderating variables, including subject area, course type, academic period, group size, class size, and experiment period. The results derived from the meta-analysis are further discussed and analyzed below.

### 5.1. Project-based learning has a positive effect on student learning outcomes

First, the combined effect value of SMD = 0.441 (*p* < 0.001) for the effect of project-based learning on learning outcomes indicates that compared to the traditional teaching model, project-based teaching has a moderately positive contribution to students’ academic achievement, thinking skills, and affective attitudes, which is consistent with the results of previous studies (Wenlan and Jiao, 2019). This is consistent with previous studies. Compared with the traditional “teacher teach-student receive-evaluate and feedback” model, project-based learning is closer to a “complete learning process” (Changming, 2020). It is a student-centered learning activity in which students show richer affective attitudes such as interest in learning and attitudes toward learning, which can positively guide students’ motivation to learn and influence their academic performance, and is naturally more effective in developing students’ emotional attitudes and values, and thinking skills.

Second, project-based learning has a significant positive effect on students’ thinking skills (SMD = 0.387, *p* < 0.001) and affective attitudes (SMD = 0.379, *p* < 0.001), indicating that the effect of project-based learning on students’ learning outcomes is not only the effect of academic performance, but also the effect of self-emotional attitudes and values, creative thinking skills, computational thinking skills, and other higher-order The impact of project-based learning on students’ learning is not only on their academic performance, but also on their self-emotional attitudes and values, creative thinking skills, computational thinking skills and other higher-order thinking skills. Project-based learning is a classroom activity that effectively develops students’ core literacies (Hongxing, 2017) and promotes the development of higher-order thinking (Weihong and Yinglong, 2019). The real value of project-based learning lies in its ability to enhance students’ higher-order thinking skills, such as creative thinking skills, problem-solving skills, and integrated application skills, by exploring real problems in small groups as a way to acquire the core concepts and principles of subject knowledge, and by posing driving questions around a topic based on real situations and students’ deep involvement in the investigation. Education for the future requires project-based learning to develop students’ 21st century skills and core literacies for their future careers and lives.

### 5.2. Moderating effects of different variables on student learning outcomes

To better analyze the impact brought by different moderating variables, this study categorized the moderating variables into four major categories: first, country region; second, curriculum, including subject categories and course types; third, teaching, including experimental period and learning periods; and fourth, experimental scale, including class size and group size. The results of the meta-analysis show as follows: (1) the application effect of project-based learning in Asia is better than that in countries in Oceania and Western Europe; (2) project-based learning has different degrees of influence on different disciplines and is better applied in the type of laboratory course; (3) in terms of the experimental period, the experimental period of 9–18 weeks is more appropriate and the application advantage of project-based learning at the high school level is more obvious; (4) project-based learning is more suitable for small-class teaching, in which the best effect is achieved when the group size is 4–5 students.

In terms of country region, the combined effect value of project-based learning is 0.358, and the application effect varies in different countries. In the Asian region, especially Southeast Asia, the effect of project-based learning is significantly better than that of Western Europe and North America. This study suggests the following reasons: First, Southeast Asian countries are relatively lagging in economic development, and industrialization and modernization are slower, so students and teachers pay more attention to practical learning methods, and project-based learning is a practice-based, problem-solving-oriented learning method that can better help them adapt and master skills and knowledge in actual work. Secondly, because the level of basic education in some Southeast Asian countries is relatively low due to various factors such as history, culture, and society, the project-based learning method can help students understand practical problems more deeply, comprehend knowledge, and enhance their hands-on and problem-solving abilities. Third, in Western European countries, students and teachers focus more on theoretical knowledge and logical thinking, individual student performance, and competition, and in countries such as Oceania, students and teachers focus more on practicality and teamwork. In Asia, however, the educational culture emphasizes a focus on discipline, order, and respect for teachers, making project-based learning more acceptable to students and parents. Students’ attitudes toward learning are also generally more serious, hard-working, and diligent, focusing on academic performance and opportunities for advancement, so students are more willing to engage in project-based learning in the hope of achieving better learning outcomes. Fourthly, in Asia, especially in East Asia, there is a strong demand for high-quality human resources, and project-based learning can cultivate students’ practical skills and innovative spirit, making them more competitive and capable of adapting to the future society.

In terms of curriculum, the combined effects of project-based learning on different subject areas and different course types were approximately equal, at 0.443 and 0.441, respectively, and the effect on student learning in engineering and technology disciplines was more significant (SMD = 0.619) and larger than the average effect, which is consistent with previous research findings that PBL is more appropriate for teaching in engineering (Kolmos and De Graaff, 2014). Facing the rapidly developing society, the traditional teaching methods seem to be unable to better develop students’ skills to meet the market demand, and the research results also show that the application effect of PBL in experimental classes (SMD = 0.498) is better than that in theoretical classes (SMD = 0.393), because PBL can give students a complete understanding of the process of a project from problem raising to problem-solving, which provides them with valuable practical experience.

From the instructional aspect, the experimental period of 9–18 weeks (SMD = 0.673) had the greatest impact on student learning effects, and the impact of project-based learning for more than 18 weeks (SMD = 0.359) was relatively low, while the results of the study showed that project-based learning had a greater impact at the high school level (SMD = 0.720), followed by elementary school, middle school, and university, a finding that supports the results of Mehmet’s study (Ayaz and Soeylemez, 2015). The moderating effect of the experimental period showed that the longer the experiment, the better the effect of about half a semester, and the project-based learning did not have a lasting and stable effect on students’ learning outcomes. Currently project-based learning is carried out more often at the primary and secondary school levels, and the teaching effect is more significant, but the application effect in universities is relatively low (SMD = 0.116), and the results of the study also indicate that the application promotion effect is most obvious in engineering and technology disciplines, so in the follow-up study, the application of project-based learning at the higher education level should be actively explored.

In terms of experimental scale, the effect of project-based learning on small class teaching (SMD = 0.483) is greater than that of medium class (SMD = 0.466) and large class (SMD = 0.106), and the teaching effect is better for group size of 4–5 people (SMD = 0.909), 8 people and above (SMD = 0.514), and 6–7 people (SMD = 0.436) in decreasing order. Therefore, project-based learning is more suitable for small-class teaching, and the number of people in the group collaborative learning is more conducive to the learning effect of around 4–5 people, which is almost consistent with the results of Wei et al. (2020) study on the effect of cooperative learning on learning effect. The relationship between class size and educational output has been discussed by a number of economists from the perspective of the economics of education, and is referred to as the “class size effect.” In small classes, teachers can spend more time on teaching and learning, each student can receive more attention from the teacher, and teachers and students can have more time to interact, thus having more opportunities to demonstrate and participate in collaborative group learning. In terms of group size, although there is no uniform standard, in general, too few or too many group members are not conducive to a higher degree of impact on the learning effect. From the research results, the best learning effect is produced by 4–5 students, with more reasonable task distribution among group members, all with a clear division of labor and sufficient interaction, which is more conducive to the formation of the group effect, thus better promoting the learning effect.

### 5.3. How does the impact of project-based learning on learning outcomes occur?

**Figure 4:**
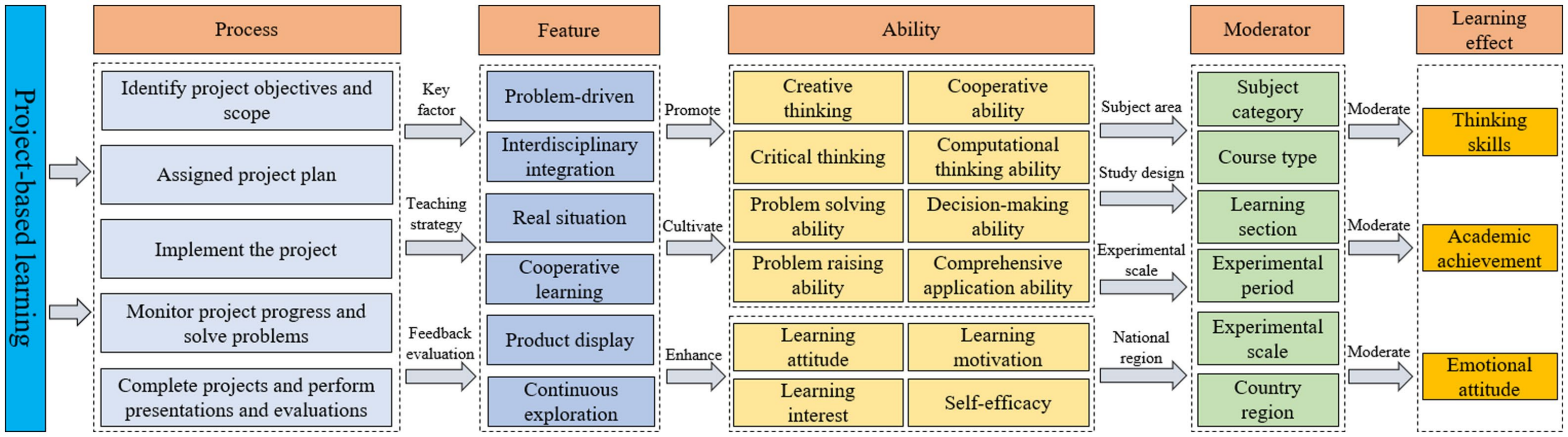
Theoretical framework for the impact of project-based learning on learning effects.

The results of the study show that project-based learning has a moderate positive contribution to learning effectiveness under different measurement measures dimensions, and how its effect occurs. The theoretical framework of the impact of project-based learning on learning effectiveness is drawn in conjunction with the specific processes and key features of project-based learning, as shown in Figure 4, and will be analyzed in the following in conjunction with the theoretical framework.

In terms of the specific process of project-based learning, it includes five steps: identifying project goals and scope, developing a project plan, implementing the project, monitoring project progress and solving problems, completing the project and presenting and evaluating it, and these steps include key activities that affect learning outcomes such as problem orientation, cooperative learning, and authenticity, which together affect students’ learning outcomes.

Specifically, project-based learning is usually oriented to real-life problems, requiring students to apply their knowledge and skills to solve problems, and the driving questions stimulate students’ interest in learning; it integrates the knowledge and skills of multiple disciplines, blending theoretical knowledge with practice and cultivating students’ creative thinking skills and comprehensive application skills; in the process of implementing projects, group members divide the work and cooperate to identify problems and After the project is completed and presented, the teacher gives timely feedback and evaluation to influence students’ attitude in project-based learning and improve the learning effect. In conclusion, the specific process and characteristics of project-based learning are the key factors to enhance students’ learning effect. Reasonable design of project characteristics and the application of different variables in project-based learning can effectively enhance students’ learning effect.

### 5.4. When is it more effective to use project-based learning?

The findings suggest that learning effects are influenced by different moderating variables, and this study suggests combining the effects of different variables for project-based learning in order to achieve the optimal effect size. For high school students in the field of engineering and technology subject areas of laboratory courses to 9–18 weeks as the experimental period, based on small class teaching, and group size of 4–5 people using the PBL method of teaching, to promote the improvement of student learning outcomes more effective. In experimental courses, the use of project-based learning can enable students to gain a deeper understanding of the principles and practical operations of experiments, increase their interest and motivation, and promote the development of their active learning and innovative thinking skills, thus improving learning outcomes. Small class teaching and group work can better meet students’ individual needs, enhance their sense of participation and belonging, and increase their interest and motivation in learning. Finally, the 9–18 weeks experimental cycle allows students to make the most of their time and explore the subject matter in depth, enabling them to gain deeper understanding and experience in their learning. It is hoped that the results of this study will provide a reference for front-line educators to carry out project-based teaching and explore more effective ways to promote learning outcomes.

## 6. Conclusion

This study conducted a meta-analysis of 66 empirical research papers on the use of project-based learning interventions for learning, and the findings provide evidence for the use of project-based learning in education to develop students’ core literacy and higher-order thinking skills, and 21st-century skills. The results show that: (1) project-based learning can significantly improve students’ learning outcomes compared with traditional teaching models; (2) the effects of project-based teaching are influenced by different moderating variables, including subject area, course type, academic period, group size, class size, and experiment period. From the perspective of countries and regions, the effect of project-based learning in Asia, especially in Southeast Asia, is significantly better than that in Western Europe and North America; from the perspective of courses, project-based learning has a more obvious effect on promoting students’ learning in engineering and technology disciplines, and the application effect in experimental classes is better than that in theory classes; from the perspective of teaching, project-based learning is more suitable for small-class teaching, in which the best effect is achieved with a group size of 4–5 students From the perspective of teaching, project-based learning is more suitable for small class teaching, and the best effect is achieved in group size of 4–5 students.

## 7. Limitation

Although our findings have important implications for educators, they still have some limitations. For example, some studies using project-based learning for teaching and learning lacked sufficient statistical information for inclusion in the analysis, and most of the studies did not provide a specific classification of learning effectiveness, limiting our ability to analyze learning effectiveness enhancement in more detail. Subsequent research can be carried out in depth in two aspects: (1) the current empirical studies on project-based learning focus on primary and secondary schools, with less research on the impact on universities and young children; with the popularity of higher education, future research can be conducted on the above research subjects; (2) taking the digital transformation of education as an opportunity to explore the integration of technology and project-based learning to better develop students’ core literacy and 21st century skills.

## Data availability statement

The original contributions presented in the study are included in the article/supplementary material, further inquiries can be directed to the corresponding author.

## Author contributions

YM: critically review the work, provide commentary, supervise and direct the writing of the draft. LZ: conceptualization, methodology, validation, quantitative data analysis, writing, review and editing. All authors contributed to the article and approved the submitted version.

## Funding

This work was supported by the Chongqing graduate education teaching reform research project (No. yjg201009), the Postgraduate Research Innovation Project of Chongqing in 2023 (No. CYS23419, No. CYS23416), and the Special Project of Chongqing Normal University Institute of Smart Education in 2023 (No. YZH23013).

## Conflict of interest

The authors declare that the research was conducted in the absence of any commercial or financial relationships that could be construed as a potential conflict of interest.

## Publisher’s note

All claims expressed in this article are solely those of the authors and do not necessarily represent those of their affiliated organizations, or those of the publisher, the editors and the reviewers. Any product that may be evaluated in this article, or claim that may be made by its manufacturer, is not guaranteed or endorsed by the publisher.
